# *ZmWRKY104* Transcription Factor Phosphorylated by *ZmMPK6* Functioning in ABA-Induced Antioxidant Defense and Enhance Drought Tolerance in Maize

**DOI:** 10.3390/biology10090893

**Published:** 2021-09-10

**Authors:** Lili Zhao, Jingwei Yan, Yang Xiang, Yue Sun, Aying Zhang

**Affiliations:** 1 College of Life Sciences, Nanjing Agricultural University, Nanjing 210095, China; 2016216025@njau.edu.cn (L.Z.); jingweiyan@njau.edu.cn (J.Y.); 2017216005@njau.edu.cn (Y.X.); sunyueacer@163.com (Y.S.); 2 State Key Laboratory of Crop Genetics and Germplasm Enhancement, Nanjing Agricultural University, Nanjing 210095, China

**Keywords:** WRKY transcription factor, mitogen-activated protein kinase (MAPK), abscisic acid (ABA), phosphorylation, antioxidant defense, drought stress, maize, *ZmWRKY104*, GRMZM2G169149, *ZmMPK6*, NP_001105238, *ZmActin*, EU952376

## Abstract

**Simple Summary:**

Current knowledge about the downstream substrate proteins of MAPKs is still limited. Our study screened a new WRKY IIa transcription factor as the substrate protein of *ZmMPK6*, and its phosphorylation at Thr-59 is critical to the role of *ZmWRKY104* in ABA-induced antioxidant defense. Moreover, overexpression *ZmWRKY104* in maize enhances the drought tolerance of transgenic plants. These findings define a mechanism for the function of *ZmWRKY104* phosphorylated by *ZmMPK6* in ABA-induced antioxidant defense and drought tolerance.

**Abstract:**

Mitogen-activated protein kinase (MAPK) cascades are primary signaling pathways involved in various signaling pathways triggered by abiotic and biotic stresses in plants. The downstream substrate proteins of MAPKs in maize, however, are still limited. Here, we screened a WRKY IIa transcription factor (TF) in maize (*Zea* *mays* L.), *ZmWRKY104*, and found that it is a substrate of *ZmMPK6*. *ZmWRKY104* physically interacts with *ZmMPK6* in vitro and in vivo. Liquid chromatography–tandem mass spectrometry (LC-MS/MS) analysis results showed that threonine-59 (Thr-59, T59) was the major phosphorylation site of *ZmWRKY104* by *ZmMPK6*. Subcellular localization analysis suggested that *ZmWRKY104* acts in the nucleus and that *ZmMPK6* acts in the nucleus and cytoplasmic membrane in the cytosol. Functional analysis revealed that the role of *ZmWRKY104* in ABA-induced antioxidant defense depends on *ZmMPK6*. Moreover, overexpression of *ZmWRKY104* in maize can enhance drought tolerance and relieve drought-induced oxidative damage in transgenic lines. The above results help define the mechanism of the function of *ZmWRKY104* phosphorylated by *ZmMPK6* in ABA-induced antioxidant defense and drought tolerance in maize.

## 1. Introduction

Abiotic stresses such as drought, salinity, oxidative stress, and temperature variations change the productivity of major crops and constitute the main cause of global crop losses [1,2]. Maize (*Zea mays* L.) is a major cereal crop species worldwide that is severely impacted by various abiotic stresses during its growth and productivity. Abiotic stresses also activate the emergence of reactive oxygen species (ROS) in plant cells. Abscisic acid (ABA) serves as a key endogenous messenger in the abiotic stress responses of plants [3], and can induce antioxidant defense-related enzymes such as ascorbate peroxidase (APX), superoxide dismutase (SOD), glutathione peroxidase (GPX) and catalase (CAT) [4]. Many signal molecules, such as calcium ion, nitric oxide (NO), and protein kinases, such as calcium-dependent protein kinase (CDPK), calcium/calmodulin-dependent protein kinase (CCaMK), and mitogen-activated protein kinase (MAPK), are involved in ABA signal response [5,6,7]. Besides, ABA can regulate plant growth, photosynthesis, and the synthesis of phenolic compounds [8,9]. Understanding ABA-induced antioxidant defense is essential for improving plant performance under abiotic stress.

The prototypical MAPK phosphorylation cascade is composed of a set of three evolutionarily conserved MAPKs, namely, MAPK kinase kinase (MAPKKK, MEKK), MAPK kinase (MAPKK, MEK), and MAPK, which play important roles in plant growth, development, and defense responses [10]. Activated MAPK phosphorylates and activates downstream substrate, such as transcription factors (TFs) at conserved amino acid residues (S/T-P motif). MAPK cascades not only play important roles in the regulation of many biological processes in plants but are also activated by drought, salinity, cold, wounding, ROS, and hormone stimuli [11]. According to their sequence homology and conserved phosphorylation motifs, MAPKs have been categorized into four major groups. Members of groups A and B have been extensively reported, and increasing knowledge about Group C MAPKs has emerged [12,13,14,15,16], but few studies have investigated the function of MAPKs belonging to group D. Group D MAPKs carry a T-D-Y phosphorylation motif. *Gossypium hirsutum* MPK16, which was the first group D MAPK gene found in cotton, is involved in drought sensitivity [12]. MPK18, *Arabidopsis* group D MAPK, involved in microtubule-related functions [17]. ZmMPK17, a maize group D MAPK gene, is in response to ABA, hydrogen peroxide, salicylic acid, jasmonic acid, ethylene, low temperature, and osmotic stress [18]. *ZmMPK6* is a member of group D of plant MAPKs and interacts with 14-3-3 proteins in vitro, but its function has not been defined [19].

As one of the largest families of transcriptional regulators in higher plants, WRKY TFs form integral components of signaling networks that modulate various plant processes [20,21]. The characteristic feature of the WRKY superfamily is the presence of a highly conserved WRKY domain containing the almost invariant WRKYGQK peptide at the N-terminus and zinc-finger motifs at the C-terminus [22]. According to the single finger motif of a small subset of WRKY proteins, WRKYs are divided into groups I, II, and III [20]. Building on the primary amino acid sequence, the WRKY group II family is further separated into IIa, IIb, IIc, IId, and IIe [23]. It has been shown that the function of most WRKY TFs in regulating transcriptional reprogramming associated with plant abiotic responses such as salt, drought, and cold. A study showed that activated expression of *AtWRKY57* could improve drought tolerance of *Arabidopsis* by elevation of ABA levels [24]. *AtWRKY46* regulated responses to osmotic/salt stress in *Arabidopsis* [25]. *TaWRKY1* and *TaWRKY33* were involved in abnormal responses to drought and ABA [26]. *AtWRKY46*, *AtWRKY54*, and *AtWRKY70*, are involved in plant growth and drought response [27]. *GmWRKY12* was highly induced by drought and salt treatments [28]. OsWRKY IIa subfamily members (*OsWRKY62*, *OsWRKY28*, *OsWRKY71*, and *OsWRKY76*) modulate innate immunity in rice [29]. Barley WRKY IIa transcripts *HvWRKY1*/*2* suppress the basal defense mechanism against virulent *Blumeria graminis*, as demonstrated by silencing and transient overexpression experiments [30,31]. *Arabidopsis WRKY18*, *WRKY40*, and *WRKY60* are involved in a complex pattern in plant responses to ABA and abiotic stresses [32]. WRKY TFs can be activated through several pathways, such as phosphorylation by mitogen-activated protein kinases (MPKs) [33,34]. Despite the functional characterization of WRKY TFs activated by MAPKs in *Arabidopsis* and rice [35,36], WRKY TFs in maize have not been studied extensively.

In the current study, we identified a WRKY IIa TF, *ZmWRKY104*, as the substrate protein of *ZmMPK6* in maize, investigated the interaction between *ZmWRKY104* and *ZmMPK6*, and analyzed the roles of *ZmWRKY104* in ABA-induced antioxidant defense and drought tolerance in maize.

## 2. Results

### 2.1. Identification and Sequence Analysis of ZmWRKY104 and ZmMPK6

The full-length coding DNA sequence (CDS) of *ZmWRKY104* (GRMZM2G169149), which encodes a polypeptide consisting of 266 amino acid residues, was obtained from the Maize GDB (https://maizegdb.org/, accessed on 1 May 2020). Conserved domain analysis revealed that *ZmWRKY104* has a highly conserved WRKYGQK motif and C2H2 (C-X5-C-X23-H-X1-H) zinc finger motif (Figure S1A). Multiple alignment shows that WRKY was highly conserved among *A. thaliana*, *O. sativa*, and *Z. mays* (Figure S1B). Phylogenetic analysis showed that *ZmWRKY104* belongs to the WRKY IIa subfamily with a single WRKY domain, and is highly homogeneous to *OsWRKY62* (Figure S2) [37]. In a previous study, we screened the possible interacting proteins of *ZmWRKY104* and identified a serine/threonine protein kinase—*ZmMPK6*. The full-length cDNA sequence of *ZmMPK6* (NP_001105238) was obtained from GenBank (https://www.ncbi.nlm.nih.gov/genbank/, accessed on 1 May 2020), which encodes a polypeptide of 557 amino acid residues and a potential S_TKc. Multiple alignment revealed that MAPK was greatly conserved among *O. sativa*, *A. thaliana*, *G. hirsutum*, *T. aestivum*, and *Z. mays*, and a phylogenetic analysis showed that *ZmMPK6* was highly homogeneous to *OsMPK15* (Figure S3B). *ZmMPK6* belongs to Group D of plant MAPKs due to the presence of the T-D-Y activation motif in the T-loop via an extended C-terminal region (Figure S3A) [19].

### 2.2. ZmWRKY104 Is a Positive Regulator in ABA-Induced Antioxidant Defense

**Figure 1:**
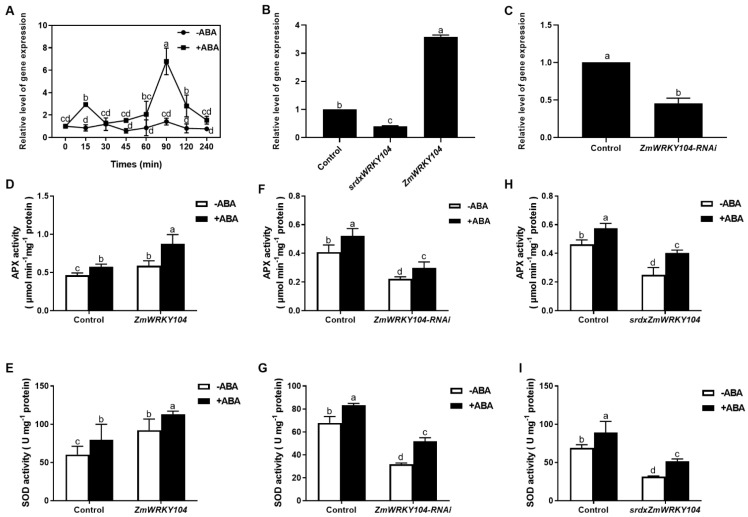
*ZmWRKY104* is related to ABA-induced antioxidant defense. (**A**) Expression levels of *ZmWRKY104* in maize leaves treated with ABA. The separated maize plants were treated with 100 µM ABA for different times. qRT-PCR detect the expression levels of *ZmWRKY104* relative to *ZmActin*; (**B**) detection of the efficiency of transiently overexpressing *ZmWRKY104* and *srdx*ZmWRKY104** in maize protoplasts; (**C**) detection of the efficiency of transiently silencing *ZmWRKY104* in maize protoplasts. Gene expression analysis of *ZmWRKY104* by RT-qPCR, with *ZmActin* as an internal control. Activities of APX (**D**) and SOD (**E**) of transiently expressing *ZmWRKY104* in protoplasts. *ZmWRKY104* (ubi: *ZmWRKY104*-mCherry) or Control (empty vector) were transfected into protoplasts. Activities of APX (**F**) and SOD (**G**) of transiently silencing *ZmWRKY104* in protoplasts. *ZmWRKY104*-RNAi or Control (distilled water) were transfected into protoplasts. Activities of APX (**H**) and SOD (**I**) of transiently expressing *srdx*ZmWRKY104** in protoplasts. *srdx*ZmWRKY104** (ubi: *ZmWRKY104*-SRDX-mCherry) or Control (empty vector) were transfected into protoplasts. Culture medium (-ABA) or 10 μM ABA (+ABA) were treated with the protoplasts for 10 min. Values are the means ± SE of three different experiments. According to Duncan’s multiple range test, means represented by the same letter did not significantly differ at *p* < 0.05.

We first assessed the expression patterns of *ZmWRKY104* in different tissues of maize. We isolated total RNA from different tissues, including roots, stems, leaves, pollen, and pistil. The results of qRT-PCR showed that *ZmWRKY104* were expressed in all tested tissues, and the expression was the highest in leaves (Figure S4). The expression of *ZmWRKY104* increased at 15 min and reached a maximum at 90 min, with a sevenfold change, and maintained for up to 240 min after treatment in maize leaves, which suggested an association of this TF with the response to ABA (Figure 1A). To investigate the part of *ZmWRKY104* in ABA-induced antioxidant defense, we determined the effect of the transient expression or silencing of *ZmWRKY104* in maize mesophyll protoplasts [38,39] on the activities of primary enzymes of APX and SOD in antioxidant defense. Firstly, we detected the efficiency of transiently overexpressing *ZmWRKY104*, silencing *ZmWRKY104*, and *srdx*ZmWRKY104** in maize protoplasts. The results showed that compared with the control, the gene expression level of *ZmWRKY104* was increased by about 3.5-fold after transient transformation (Figure 1B). After transient transformation of *ZmWRKY104*-RNAi (Figure 1C) and *srdx*ZmWRKY104** (Figure 1B), compared with the control, the gene expression level decreased by about 50%, indicating that the transient transformation of protoplasts can achieve the expected effect. As shown in Figure 1D,E, the activities of APX and SOD after transient expression of *ZmWRKY104* were significantly higher than those in the control, and ABA treatment further enhanced these increased activities. On the contrary, transient silencing of *ZmWRKY104* significantly reduced its activities compared with the control, and ABA treatment could not return these decreased activities to baseline levels (Figure 1F,G). WRKY TFs have functional redundancy [40]. To validate the function of *ZmWRKY104*, chimeric repressor silencing technology was used [7,24,25]; we fused **ZmWRKY104*-*SRDX-mCherry to the exogenous EAR motif repression domain SRDX and transformed the product (*srdx*ZmWRKY104**) into maize protoplasts. Similarly, transient expression of *srdx*ZmWRKY104** also resulted in significant decreases in the activities of APX and SOD, and ABA treatment could not return their activities to the control levels (Figure 1H,I). In summary, all the above-described results provide clear facts that *ZmWRKY104* is a positive regulator in ABA-induced antioxidant defense.

### 2.3. ZmWRKY104 and ZmMPK6 Function in ABA-Induced Antioxidant Defense

**Figure 2:**
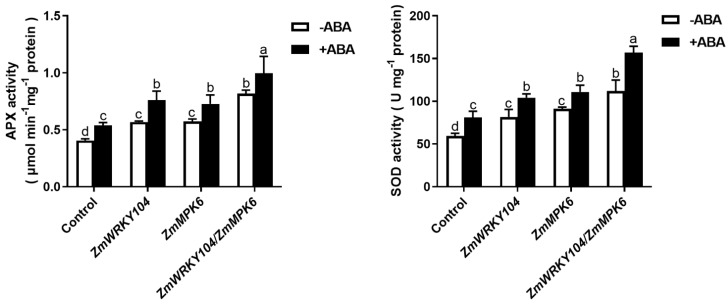
Overexpression of *ZmWRKY104* and *ZmMPK6* in protoplasts enhances the activities of ABA-induced antioxidant defense enzymes in maize. The activities of APX and SOD in protoplasts transiently expressing ubi: *ZmWRKY104*-mCherry alone (*ZmWRKY104*), ubi: *ZmMPK6*-mCherry alone (*ZmMPK6*) or ubi*: *ZmWRKY104**-mCherry and ubi: *ZmMPK6*-mCherry (*ZmWRKY104*/*ZmMPK6*) simultaneously in maize mesophyll protoplasts. Culture medium (-ABA) or 10 μM ABA (+ABA) were treated with the protoplasts for 10 min. Values are the means ± SE of three different experiments. According to Duncan’s multiple range test, means represented by the same letter did not significantly differ at *p* < 0.05.

Our previous research reported that MAPK was related to ABA-induced antioxidant defense [6]. Thus, we wondered whether **ZmWRKY104* and *ZmMPK6** function together in ABA-induced antioxidant defense. As shown in Figure 2, the activities of APX and SOD after transient expression of *ZmWRKY104* or *ZmMPK6* alone were significantly increased, and these increased activities were further enhanced by ABA treatment. Compared with *ZmWRKY104* or *ZmMPK6* alone, co-expression of *ZmWRKY104* and *ZmMPK6* further increased the activities. The activities of APX and SOD were also further enhanced by treatment with ABA. These results suggest that co-expression of *ZmWRKY104* and *ZmMPK6* improves ABA-induced antioxidant defense in maize.

**Figure 3:**
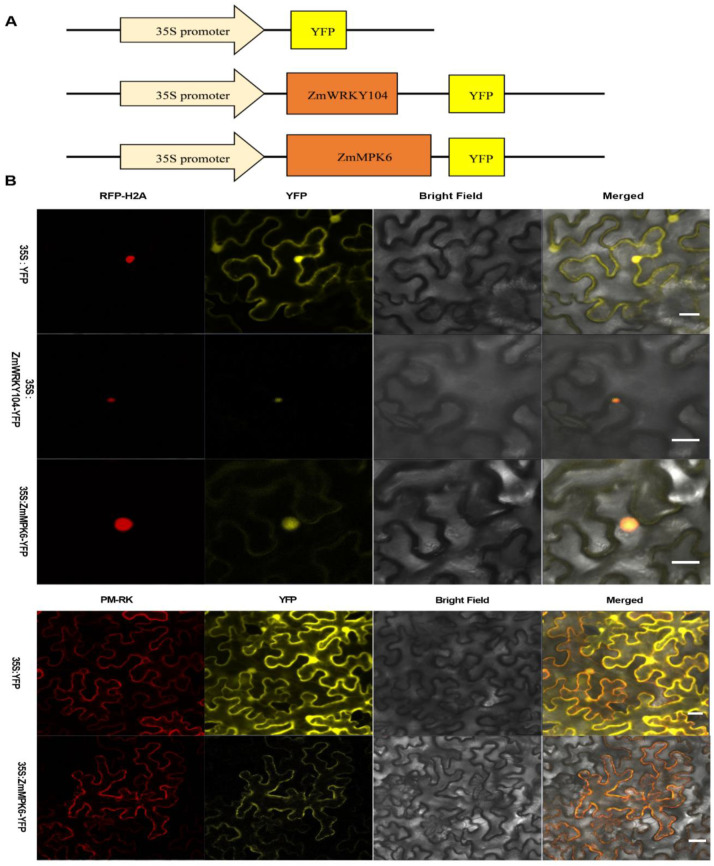
Subcellular localization of *ZmWRKY104* and *ZmMPK6* in tobacco leaf epidermal cells. (**A**) Vector diagram of 35S: YFP, 35S: **ZmWRKY104*-*YFP, and 35S: *ZmMPK6*-YFP; (**B**) constructs carrying 35S: YFP, 35S: **ZmWRKY104*-*YFP, and 35S: *ZmMPK6*-YFP were transformed into tobacco leaves by agroinfiltration. Transfected leaves were observed by a confocal laser scanning microscope. Plasma membranes are shown with PM-RK (red). Nuclei are shown with RFP-H2A (red). Experiments were repeated three times with similar results. Scale bars = 20 μm.

Moreover, we investigated the subcellular localizations of *ZmWRKY104* and *ZmMPK6*. *ZmWRKY104* and *ZmMPK6* fused to yellow fluorescent protein (YFP) were transiently expressed by *Agrobacterium* infiltration in tobacco leaves. Subcellular localization results showed that *ZmWRKY104* was specifically located in the nucleus of tobacco mesophyll cells, and *ZmMPK6* was specifically located in the cytoplasmic membrane and nucleus of tobacco mesophyll cells (Figure 3).

### 2.4. ZmMPK6 Interacts with and Phosphorylates ZmWRKY104

**Figure 4:**
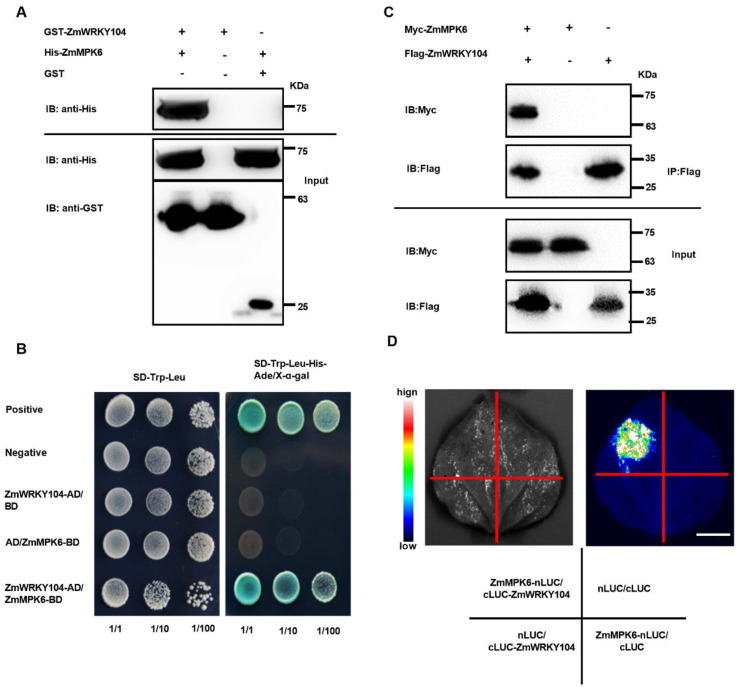
The interaction between *ZmWRKY104* and *ZmMPK6*. (**A**) Pull-down assay. GST or GST-*ZmWRKY104* fusion protein was incubated with His-*ZmMPK6* in GST beads. His-*ZmMPK6* was detected using an antibody of anti-His by Western blot. GST-*ZmWRKY104* and GST were detected using an anti-GST antibody by Western blot. Molecular masses are marked on the right; (**B**) Y2H assay. AD-vector and BD-vector were Y2H vectors with no insert. To test the interaction, the medium of SE-Trp-Leu-His-Ade/X-α-gal was used. pGBKT7-53/pGADT7-T was used as a positive control, and pGBKT7-*lam*/pGADT7-T was used as a negative control; (**C**) Co-IP assay. 35S:Myc-*ZmMPK6* and 35S:Flag-*ZmWRKY104* were co-expressed in four-week-old *Nicotiana benthamiana* leaves. Proteins were immunoprecipitated (IP) with Flag antibody and analyzed by immunoblot (IB) using anti-Flag and anti-Myc antibodies. Molecular masses are marked on the right; (**D**) LCI assay. *ZmMPK6* was fused to nLUC, and *ZmWRKY104* was fused to cLUC. *ZmMPK6*-nLUC and cLUC-*ZmWRKY104* were then co-expressed in *Nicotiana benthamiana* leaves. nLUC/cLUC-*ZmWRKY104*, *ZmMPK6*-nLUC/cLUC, and nLUC/cLUC were used as negative controls. Luciferase signals were captured using the Tanon 5200 image system. Scale bar = 1 cm. All experiments were repeated at least three times with similar results.

To identify *ZmWRKY104* as a target of *ZmMPK6*, a GST pull-down assay was first used. Full-length *ZmWRKY104* fused to GST protein and full-length *ZmMPK6* tagged with poly-His. His-tagged *ZmMPK6* was maintained on beads with immobilized GST-*ZmWRKY104* but not with immobilized GST (Figure 4A), which suggests that *ZmMPK6* interacted with *ZmWRKY104* in vitro. Subsequently, we performed Y2H assays to further identify the interaction in vitro. Full-length *ZmWRKY104* was inserted into the pGADT7 (AD) vector, and *ZmMPK6* was inserted into the PGBKT7 (BD) vector. Both vectors were then transformed and selected on SD/Trp/Leu/His/Ade with X-α-galactosidase, and the interaction between *ZmWRKY104* and *ZmMPK6* was observed (Figure 4B). We then performed Co-IP to validate the interaction between *ZmWRKY104* and *ZmMPK6* in *Nicotiana benthamiana* leaves. Proteins were immunoprecipitated with protein-A/G agarose beads and analyzed by immunoassay using anti-Myc and anti-Flag antibodies. Immunoblot (IB) analyses using an anti-Myc antibody showed an interaction between Flag-*ZmWRKY104* and Myc-*ZmMPK6* (Figure 4C). An LCI assay in tobacco leaves was then performed to further confirm the interaction between *ZmWRKY104* and *ZmMPK6*, and the results showed that the *ZmWRKY104* protein could interact with the *ZmMPK6* protein (Figure 4D). Thus, the above results indicated that *ZmMPK6* interacts with *ZmWRKY104*.

**Figure 5:**
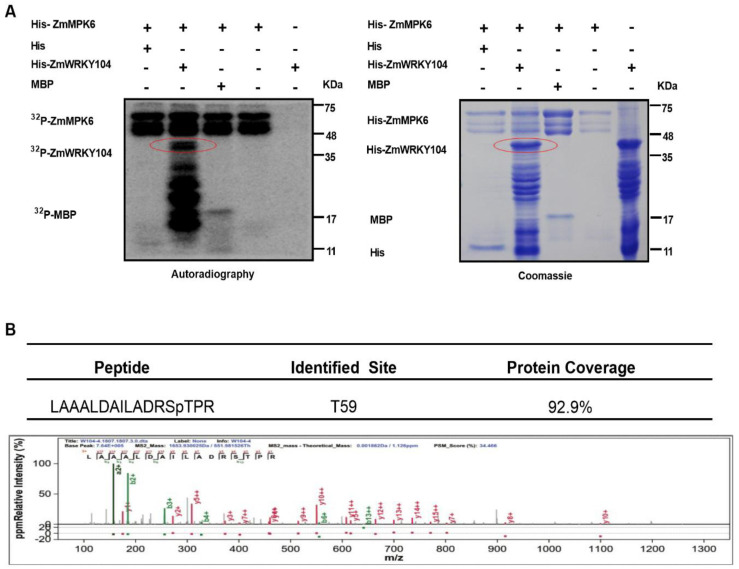
*ZmMPK6* phosphorylates *ZmWRKY104* in vitro. (**A**) *ZmWRKY104* can be phosphorylated by *ZmMPK6* in vitro. His-*ZmMPK6*, His-*ZmWRKY104*, and His proteins were expressed in *E. coli*. In vitro kinase assays were performed using the purified proteins. The substrate of His-*ZmWRKY104* or maltose binding protein (MBP) was used for an in-gel kinase assay. Images show autoradiography (left panel) and the corresponding Coomassie staining (right panel). Molecular mass markers in kilodaltons are shown on the right. Experiments were repeated at least three times with similar results. (**B**) Phosphorylation site(s) analysis of *ZmWRKY104* by LC-MS/MS.

To examine which region(s) of *ZmWRKY104* mediates its binding to *ZmMPK6*, we generated two truncations, *ZmWRKY104*^1-85AA^ and *ZmWRKY104*^86^*^-^*^266AA^ (Figure S5A), and tested these truncations both in vitro and in vivo through GST pull-down and LCI assays. As shown in Figure S5B,C, the interaction region was located at the 1–85 AA region at the N-terminus of *ZmWRKY104*. To verify that *ZmMPK6* phosphorylates *ZmWRKY104*, we performed an in vitro gel kinase assay. Results showed that *ZmMPK6* has autophosphorylation and substrate phosphorylation activity and can phosphorylate myelin basic protein (MBP) and *ZmWRKY104* (Figure 5A). Liquid chromatography–tandem mass spectrometry (LC-MS/MS) analysis results showed that Threonine-59 (Thr-59, T59) was the major phosphorylation site of *ZmWRKY104* by *ZmMPK6* (Figure 5B).

### 2.5. ZmMPK6 Phosphorylation of ZmWRKY104 Plays a Key Role in ABA-Induced Antioxidant Defense

**Figure 6:**
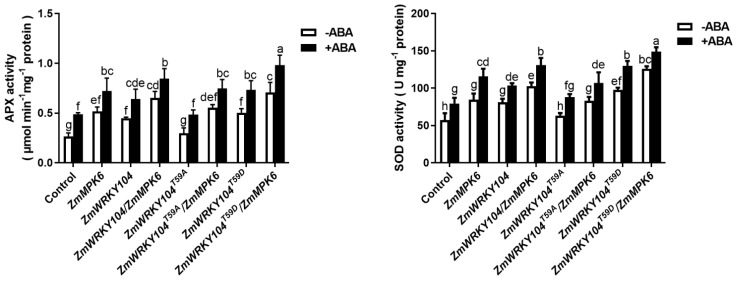
The effects of the Thr-59 site of *ZmWRKY104* in ABA-induced antioxidant defense. Ubi: **ZmWRKY104*-*mCherry alone (*ZmWRKY104*), ubi: *ZmMPK6*-mCherry alone (*ZmMPK6*) ubi: **ZmWRKY104*^T59A^*-mCherry alone (**ZmWRKY104*^T59A^*), ubi: **ZmWRKY104*^T59D^*-mCherry alone (**ZmWRKY104*^T59D^*); ubi: **ZmWRKY104*-*mCherry and ubi: *ZmMPK6*-mCherry (*ZmWRKY104*/*ZmMPK6*) simultaneously, ubi: **ZmWRKY104*^T59A^*-mCherry and ubi: *ZmMPK6*-mCherry (**ZmWRKY104*^T59A^*^/^*ZmMPK6*) simultaneously, and ubi: **ZmWRKY104*^T59D^*-mCherry and ubi: *ZmMPK6*-mCherry (**ZmWRKY104*^T59D^*/*ZmMPK6*) simultaneously were transiently expressing in protoplasts. Culture medium (-ABA) or 10 μM ABA (+ABA) were treated with the protoplasts for 10 min. Values are the means ± SE of three different experiments. According to Duncan’s multiple range test, means represented by the same letter did not significantly differ at *p* < 0.05.

To identify whether the Thr-59 site is the major site of *ZmWRKY104*, we mutated the T59 site of *ZmWRKY104* to non-phosphorylated alanine (T59A) and simulated phosphorylated state aspartic acid (T59D) for an in vitro gel kinase assay. Results showed that *ZmWRKY104*^T59A^ could be weakly phosphorylated by *ZmMPK6*, indicating that the Thr-59 site of the *ZmWRKY104* protein blocked most of the phosphorylation of *ZmWRKY104* by *ZmMPK6* (Figure S6). To explore the role of the Thr-59 site of *ZmWRKY104* in ABA-induced antioxidant defense, the maize transient expression system of protoplasts was used to test its effect on the activities of APX and SOD. As shown in Figure 6, compared with the control, transient expression of **ZmWRKY104*^T59A^* alone did not significantly influence the activities, whereas transient expression of **ZmWRKY104*^T59D^* alone significantly increased the activities. The activities in **ZmWRKY104*^T59A^*/*ZmMPK6*-co-expressed protoplasts were equal to those found in protoplasts expressing *ZmMPK6* alone. Compared with the control, co-expression of **ZmWRKY104*^T59D^*/*ZmMPK6* significantly increased the activities, and ABA treatment further increased these activities (Figure 6). These data clearly suggest that the phosphorylation of *ZmWRKY104* at Thr-59 by *ZmMPK6* plays a key role in ABA-induced antioxidant defense.

### 2.6. ZmWRKY104 Overexpression Enhances Drought Tolerance in Transgenic Maize Plants

**Figure 7:**
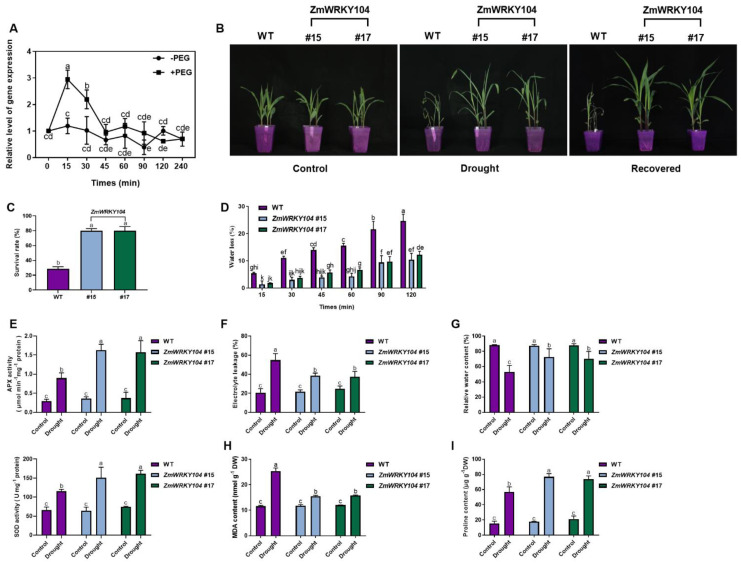
The overexpression of *ZmWRKY104* confers drought tolerance in transgenic maize plants. (**A**) PEG induces the upregulation of *ZmWRKY104* expression in the leaves of maize plants. Expression analysis of *ZmWRKY104* in leaves of maize plants exposed to 10% (*w*/*v*) polyethylene glycol (PEG6000) treatments for various times as indicated. The expression level relative to *ZmActin* was analyzed by qRT-PCR. (**B**) Phenotype of *ZmWRKY104* in transgenic maize. Seedlings were subjected to native drought conditions after the three-leaf stage (water was withheld) for 10 days and then rewatered using normal protocols for 3 days. During the experiment, control plants were watered every day. The drought test was repeated 3 times, at least 35 plants were used for each individual line, and a representative picture was displayed. Scale bars = 7 cm. (**C**) Survival rate of maize plants after rewatering for 3 days. (**D**) Analysis of water retention in *ZmWRKY104*-OE transgenic lines. Maize leaves isolated from transgenic and WT seedlings were positioned for 2 h at 25 °C, and the percentage of water loss at different time points was recorded. (**E**) Analysis of the activities of APX and SOD of *ZmWRKY104*-OE transgenic lines under drought stress. The activities were testing of the seedling’s leaves treated with Control (distilled water) or drought. (**F**) Electrolyte leakage; (**G**) the relative water content, (**H**) The MDA contents and (**I**) The proline contents were determined under drought stress and in normal-watered control plants in the leaves of *ZmWRKY104*-OE transgenic plants and WT plants. Values are the means ± SE of three different experiments. According to Duncan’s multiple range test, means represented by the same letter did not significantly differ at *p* < 0.05.

We used PEG6000 to imitate drought stress to investigate the expression of *ZmWRKY104* in maize plants. Results revealed that *ZmWRKY104* was powerfully induced at 15 min by PEG (Figure 7A). The above results indicate that *ZmWRKY104* was induced by drought stress.

To further understand the part of *ZmWRKY104* in drought stress, we generated transgenic maize plants under the control of the *ubiquitin* promoter via *Agrobacterium tumefaciens*-mediated transformation. We selected two independent lines (*#15* and *#17*; Figure S7) that exhibited similar *ZmWRKY104* gene and protein levels for further analysis. Compared with wild-type (WT) lines, **ZmWRKY104*-*OE transgenic lines displayed no morphological changes under normal growth conditions (Figure 7B). After natural drought, the wild-type lines were restricted, and the leaves were curled and yellow, while the *ZmWRKY104*-OE transgenic lines grew better, and the leaves were stretched; after rewatering, the leaves of the *ZmWRKY104*-OE transgenic lines spread out evenly, while the wild-type lines only partially survived and could not fully restore the flat state (Figure 7B). Under drought stress, compared with the WT lines, the *ZmWRKY104*-OE transgenic lines had higher survival rates (Figure 7C), leaf water retention capacity (Figure 7D), and activity of antioxidant defense enzymes (Figure 7E). In addition, the electrolyte leakage rate (Figure 7F) and malondialdehyde (MDA) content (Figure 7H) of the *ZmWRKY104*-OE transgenic lines were lower than those of the wild-type lines; the relative water content (Figure 7G) and accumulated proline content (Figure 7I) were higher than those of the wild-type lines. The above results indicate that *ZmWRKY104* enhances drought stress tolerance.

## 3. Discussion

TFs play key roles in plants, allowing them to adjust to new environmental factors, such as drought. As one of the largest families of TFs in higher plants, WRKY families play crucial roles in response to various stresses [12,14,26]. More and more evidence show that WRKY protein physically interacts with a variety of proteins, and these proteins play a role in controlling or regulating various processes. MAP kinase is one of the main proteins that interact with WRKY TFs [41,42]. The MAP kinase pathway is related to regulate the activities of WRKY33 to manage plant defense responses [43]. The cascade may act as a molecular hub, integrating different signaling networks of WRKY TFs with various upstream proteins. The fact that most WRKYs are distinguishingly regulated by bacterial pathogens or SA treatment further confirms this, which also activates the MPK3/6 pathway [44]. In this study, we demonstrate that *ZmWRKY104*, which belongs to group IIa of the WRKY superfamily, is a positive component of the ABA-induced antioxidant defense (Figure 1).

The function of TFs or their related proteins could be regulated by MAPK of its phosphorylation [45]. In defense signaling, some studies have shown that WRKY protein is associated with defense-induced MAPK signaling cascade. The NtMEK2-SIPK/WIPK signaling pathway is involved in regulating the expression of defense genes in tobacco [41]. Here, our data show that *ZmMPK6*-phosphorylated *ZmWRKY104* increases ABA-induced antioxidant defense in maize, and phosphorylation at the Thr59 position plays an important role in this process (Figure 5 and Figure 6). Therefore, the modulation of WRKY protein phosphorylated by MAPK seems to be a common phenomenon for regulating plant defense responses. 

Subcellular localization experiments revealed that *ZmWRKY104* focuses on the nucleus (Figure 3), which accords with the truth that it acts as a TF, potentially modulating the transcription of defense-related genes. However, how Thr-59 phosphorylation affects the DNA binding of *ZmWRKY104* is unclear. In addition, the cascade upstream of *ZmMPK6* in ABA signaling in maize also needs to be further explored.

Overexpression of *ZmWRKY104* modified the drought tolerance of maize plants (Figure 7), indicating that this gene is a positive regulator of drought resistance. Detailed analysis revealed that *ZmWRKY104* overexpression alleviates the degree of oxidative damage in response to drought in maize. First, *ZmWRKY104* overexpression reduced the electrolyte leakage, which is an immediate stress marker of damage in cell membrane under drought conditions [46]. Second, *ZmWRKY104* overexpression lines accumulated higher proline than WT plants. The accumulation of high levels of proline was beneficial to plant resistance to environmental stress [47]. Third, *ZmWRKY104* overexpression lines possessed lower MDA contents, and MDA is typically used as an important indicator of oxidative damage under environmental stresses [48]. Fourth, *ZmWRKY104* overexpression lines had higher relative water content than WT plants, which is necessary for plants to survive under drought conditions. Furthermore, the alleviation of oxidative damage might be caused by enhanced antioxidant defense.

In summary, our data show that *ZmWRKY104* interacts with and is phosphorylated by *ZmMPK6*, Thr-59 phosphorylation is critical to the role of *ZmWRKY104* in ABA-induced antioxidant defense, and overexpression of *ZmWRKY104* enhances drought tolerance and alleviates drought-induced oxidative damage.

## 4. Materials and Methods

### 4.1. Plant Materials and Treatments

In this study, we use maize (*Zea mays* L., cv. B73) and tobacco (*Nicotiana benthamiana*) seeds. The maize seed was sown in trays placed in an environmental chamber with a photosynthetically active radiation (200 µmol m^−2^ s^−1^), a temperature (22–28 °C), and a 14 h/10 h (day/night) photoperiod and watered every 2 days [49].

Maize plants were grown under dark conditions at 25 °C, and the second leaf was fully extended to extract protoplasts. We cut the plant from the base of the stem and put it in distilled water for 2 h to relieve the wound pressure. After this treatment, we put the cut end of the stem into a beaker wrapped in aluminum foil, which contained a solution of 100 µM ABA and 10% (*w*/*v*) polyethylene glycol (PEG6000) solution. Plants immersed in distilled water for the entire period under the same conditions served as controls. After the treatment, subsequent leaves of the separated maize plants were sampled and immediately frozen in liquid nitrogen.

Tobacco seeds were sown in a soil pot with a photosynthetically active radiation (120 µmol m^−2^ s^−1^), a temperature (23 °C), and a photoperiod of 16 h/8 h (day/night) and watered every two days. After 4 weeks of growth, we use the plants for subsequent experiments.

### 4.2. GST Pull-Down Assay

GST, GST-*ZmWRKY104*, and truncated (GST-*ZmWRKY104*^1^*^-^*^85AA^ and GST-*ZmWRKY104*^86^*^-^*^266AA^) fusion proteins were maintained, immobilized, on Magnet GST particles (Promega, Madison, USA) and then incubated with His-tagged *ZmMPK6* in binding buffer (10 mM KCl, 4.2 mM Na_2_HPO_4_, 140 mM NaCl, 2 mM KH_2_PO_4_, and 10% bovine serum albumin, pH 7.2) with gentle shaking for 2 h at 4 °C. Then, washing buffer (140 mM NaCl, 4.2 mM Na_2_HPO_4_, 2 mM KH_2_PO_4_, and 10 mM KCl, pH 7.2) wash the beads more than 3 times, and use the 1 × SDS loading buffer (50 mM Tris-HCl, 2% SDS, 0.1% bromophenol blue, 10% glycerol, and 10 mM dithiothreitol (DTT)) to elute the pulled down proteins by boiling, use the 12% SDS-PAGE gels to separate them, use an anti-GST antibody (Abmart, Shanghai, China) or anti-His antibody (Abmart) followed by a goat anti-mouse IgG HRP-conjugated secondary antibody (Abmart).to analyze.

### 4.3. Yeast Two-Hybrid (Y2H) Assay

According to the yeast protocol handbook (Clontech, Shiga, Japan), Y2H assays were performed. *ZmWRKY104* was cloned into pGADT7 using *EcoRI*/*BamHI* sites, *ZmMPK6* was cloned into pGBKT7 using *BamH*/*PstI* sites, and Table S1 shows the primers. The prey vector was converted into Y187 of yeast strain, and the bait vector was transformed into Y2HGold of yeast strain using the lithium acetate method. The prey and bait strains were mated and spread on stringent selective medium plates containing X-α-gal (40 μg mL^−1^), then incubated for 3–5 days at 30 °C and verified every day.

### 4.4. Co-Immunoprecipitation (Co-IP) Assay

*ZmWRKY104* or *ZmMPK6* were fused to Flag or Myc tags cloned into 1300-221 vectors using *KpnI*/*BamHI* sites or *BstBI*/*BamHI* sites. Table S1 shows the primers. 35S*: Myc-*ZmMPK6**, 35S*: Flag-*ZmWRKY104**, 35S*: Myc-*ZmMPK6**, and 35S*: Flag-*ZmWRKY104** were expressed in *Nicotiana benthamiana* leaves. After incubation for 3 days, proteins were extracted from the leaves with buffer as described previously and centrifuged at 4°C at 10,000 g for 30 min [6]. The soluble proteins were incubated with an anti-Flag (Abmart) or anti-Myc antibody (Abmart) bound to IP buffer at 4 °C for 3 h. We washed the beads with immunoprecipitation buffer 3 times and boiled for 8 min in 1 × SDS buffer to elute proteins. After centrifugation, the supernatant was analyzed with anti-MyC antibody (Abmart) by immunoblotting.

### 4.5. Luciferase Complementation Imaging (LCI) Assay

We fused full-length and truncated sequences of *ZmWRKY104* to the pC1300-cLUC vector utilizing *KpnI*/*BamHI* sites, and fused full-length sequences of *ZmMPK6* to the pC1300-nLUC vector utilizing *SacI*/*BamHI* sites. Table S1 shows the primers. Then we transformed them into GV3101 of *Agrobacterium tumefaciens* strain and incubated under dark conditions at 30 °C for 2–3 days, and selected the positive clone and incubated in yeast extract broth (YEB) liquid medium for 16 h at 28 °C [49]. The bacteria were collected in 2 mL tubes and centrifuged at 5000 rpm for 5 min at 25 °C. Then we removed the supernatant and resuspended the bacteria in buffer (10 mM MgCl_2_, 10 mM MES, pH 5.7, and 100 μM acetosyringone) to a final OD600 of 0.5. After 3 to 5 h, we injected the bacteria into *Nicotiana benthamiana* leaves, and 72 h after injection, we sprayed with 1 mM D-Luciferin (Thermo Fisher Scientific, Waltham, MA, USA) at the abaxial sides of the leaves. The leaves were then stored for 30 min in the dark, and we used a camera (Tanon 5200 Multi, Tanon Biomart, Beijing, China) to capture the signal of LUC.

### 4.6. Expression and Purification of Recombinant Proteins

*ZmWRKY104* and its truncated mutants (*ZmWRKY104^1-85AA^* and **ZmWRKY104*^86-266AA^*), were amplified, cloned into the vector of *pGEX-4T-1* with a GST tag, and expressed in BL21 (DE3) of *E. coli* strain. The expression of plasmids or the empty vector was induced for 6 h at 28 °C with isopropyl β-D-1-thiogalactopyranoside (IPTG) of 0.2 mM in LB broth on a shaker at 160 rpm. Full-length **ZmWRKY104*, *ZmWRKY104*^T59A^* or **ZmWRKY104*^T59D^* was transformed into BL21 (DE3) of *E. coli* with the His-tagged expression vector pET-30a, and induced by incubation for 6 h at 22 °C with 0.2 mM IPTG. Full-length *ZmMPK6* was cloned into the pET-30a vector in frame with the His tag, transformed into BL21 (DE3) of *E. coli* strain, and induced by incubation for 6 h at 26 °C with 0.5 mM IPTG.

### 4.7. In Vitro Kinase Assay

Recombinant His-*ZmWRKY104*, His-*ZmWRKY104*^T59A^ or His-*ZmWRKY104*^T59D^ was incubated with 0.01 mg mL^−1^ *ZmMPK6* protein in buffer of phosphorylation (20 mM MgCl_2_, 25 mM Tris-HCl, pH 7.5, 2 mM MnCl_2_, and 1 mM DTT) for 30 min at 30 °C in a final volume of 50 μL including 1 μg of MBP, 10 μM ATP, and 10 μCi [γ-^32^P] ATP. We added SDS sample buffer to stop the reaction and boiled the product for 10 min in a boiling water bath and resolved by 12% SDS-PAGE. We removed the unincorporated [γ-^32^P] ATP by washing with 5% trichloroacetic acid (*w*/*v*) and 1% sodium pyrophosphate (*w*/*v*) more than three times. The phosphorylated substrates were visualized by autoradiography.

### 4.8. Isolation of Total RNA and qRT-PCR Analysis

The analysis was performed as performed as described previously [7]. The primers are described in detail in Table S1.

### 4.9. Vector Construction and In Vitro Synthesis of Double-Stranded RNA

The construction and synthesis were performed as described previously [7]. The primers are given in Table S1.

### 4.10. Protoplast Preparation and Transfection with DNA Constructs or dsRNAs

The experiments were performed as indicated previously [7]. The transformed protoplasts were then incubated overnight at 25 °C in the dark in culture solution, treated for 10 min with or without 10 μM ABA, and utilized for the subsequent analysis.

### 4.11. Site-Directed Mutagenesis

For the mutation of *ZmWRKY104*, we utilized the M5 HiPer Site-Directed Mutagenesis Kit (Mei5bio, Beijing, China) based on the manufacturer’s instructions. Table S1 shows the DNA oligonucleotides sequences.

### 4.12. Subcellular Localization

Tobacco leaves were transfected with GV3101 of *Agrobacterium* strain carrying the fusion construct of 35S*: *ZmWRKY104*-YFP*, the 35S*: *ZmMPK6*-YFP* fusion construct, or 35S*: YFP*. Yellow fluorescence was observed as indicated previously under a Zeiss LSM710 device [7]. The nucleus was stained with a nuclear marker (RFP-H2A), and the plasma membrane was stained with a membrane marker (PM-RK) [50].

### 4.13. Antioxidant Enzyme Assay

The antioxidant enzyme assays were performed as indicated previously described [51,52,53].

### 4.14. Mass Spectrometry Analysis

The His-*ZmWRKY104* fusion protein was reacted with the His-*ZmMPK6* fusion protein in kinase assay buffer in vitro with ATP. Then we enriched the phosphorylated *ZmWRKY104* and digested by trypsin followed by mass spectrometry/liquid chromatography tandem mass spectrometry (LC-MS/MS) analysis as indicated previously [7]. The results of mass spectrometry were analyzed using the software pFind Studio (http://pfind.ict.ac.cn/software/pLink/index.html, accessed on 23 Jul 2020).

### 4.15. Generation of Transgenic Maize Plants

Insert *ZmWRKY104* into the *HindIII/KpnI* sites of the pCUN-N-HF vector with *ubiquitin* promoter; Table S1 shows the primers. We used the maize inbred line B73 for genetic transformation. The recombinant vector was introduced into maize by LBA4404 of *Agrobacterium tumefaction* strain via maize shoot tip transformation [54,55]. *Agrobacteria* containing the recombinant strain were cultured separately and then mixed in infiltration buffer to a final OD600 between 0.65 and 0.75. The mixture of *Agrobacterium* cultures were infiltrated with young maize stems by a vacuum pump (0.045 to 0.060 MPa) for 3 to 5 min and then sown in pots containing a 1:1 mixture of organic nutrient soil vermiculite. The pots were placed in an environmental chamber with 22–28 °C, photosynthetically active radiation (200 µmol m^−2^ s^−1^), and a 14 h/10 h (day/night) photoperiod and watered every 3 days. The positive plants were identified by PCR analysis and then transferred to the greenhouse for planting and harvesting, and Table S1 shows the primers. qRT-PCR analyzed the expression of *ZmWRKY104* in transgenic lines, and two independent homozygous T2 lines, **ZmWRKY104* #15* and **ZmWRKY104* #17*, were selected for subsequent experiments.

### 4.16. Drought Tolerance and Oxidative Damage Analysis

After 4 weeks of growth, seedlings were subjected to native drought conditions (water was withheld) for 10 days and then rewatered using normal protocols for 3 days. The seedling phenotype was photographed, and the survival rate was calculated. The experiments were repeated 3 times, each individual line with more than 35 plants employed in every repeated experiment, and one typical image is shown. The relative water content (RWC), malondialdehyde (MDA) levels, electrolyte leakage, the content of proline (Pro), and APX and SOD activities were measured as previously described [7,56,57,58].

### 4.17. Phylogenetic Analysis

To construct a phylogenetic tree, the sequences of plant WRKY proteins and MAPK proteins were retrieved from the NCBI database and aligned using the ClustalW program, a neighbor-joining phylogenetic tree of these sequences was then constructed, and bootstrap analysis was performed with 500 iterations using the MEGA7 program [52,59].

## 5. Conclusions

In this study, we identified a WRKY IIa TF, *ZmWRKY104*, as the substrate protein of *ZmMPK6* in maize. Liquid chromatography–tandem mass spectrometry (LC-MS/MS) analysis results showed that threonine-59 (Thr-59, T59) was the major phosphorylation site of *ZmWRKY104* by *ZmMPK6*. Functional analysis revealed that the role of *ZmWRKY104* in ABA-induced antioxidant defense depends on *ZmMPK6*. Genetic analysis showed that overexpression of *ZmWRKY104* in maize can improve drought tolerance and alleviate drought-induced oxidative damage in transgenic plants.

## Data Availability

Not applicable.

## Supplementary Materials

The following are available online at https://www.mdpi.com/article/10.3390/biology10090893/s1**, Figure S1**. Multiple alignment of *ZmWRKY104* with other orthologs in rice, *Arabidopsis*. Figure S2. Phylogenetic relationships of *ZmWRKY104* with other orthologs in rice, *Arabidopsis*. Figure S3. Multiple alignment and phylogenetic relationships of *ZmMPK6* with other orthologs in rice, *Arabidopsis*, wheat, cotton. Figure S4. RT-qPCR analysis of *ZmWRKY104* expression in different tissues of maize, Figure S5. *ZmMPK6* interacts with the truncations of *ZmWRKY104*, Figure S6. Phosphorylation of *ZmWRKY104*^T59A^ and *ZmWRKY104*^T59D^ by *ZmMPK6* in vitro, Figure S7. Analysis the gene expression and protein level of the *ZmWRKY104* transgenic lines, Table S1: PCR Primers Used.
