# Supplementary material for: ZmWRKY104 Transcription Factor Phosphorylated by ZmMPK6 Functioning in ABA-Induced Antioxidant Defense and Enhance Drought Tolerance in Maize
Source: Biology (Basel). 2021 Sep 10;10(9):893. doi: 10.3390/biology10090893 (PMC8467104; doi:10.3390/biology10090893)
Supplement: Supplementary file 1 [file biology-10-00893-s001.zip › biology-1326751-supplementary.pdf]

SUPPORTING INFORMATION

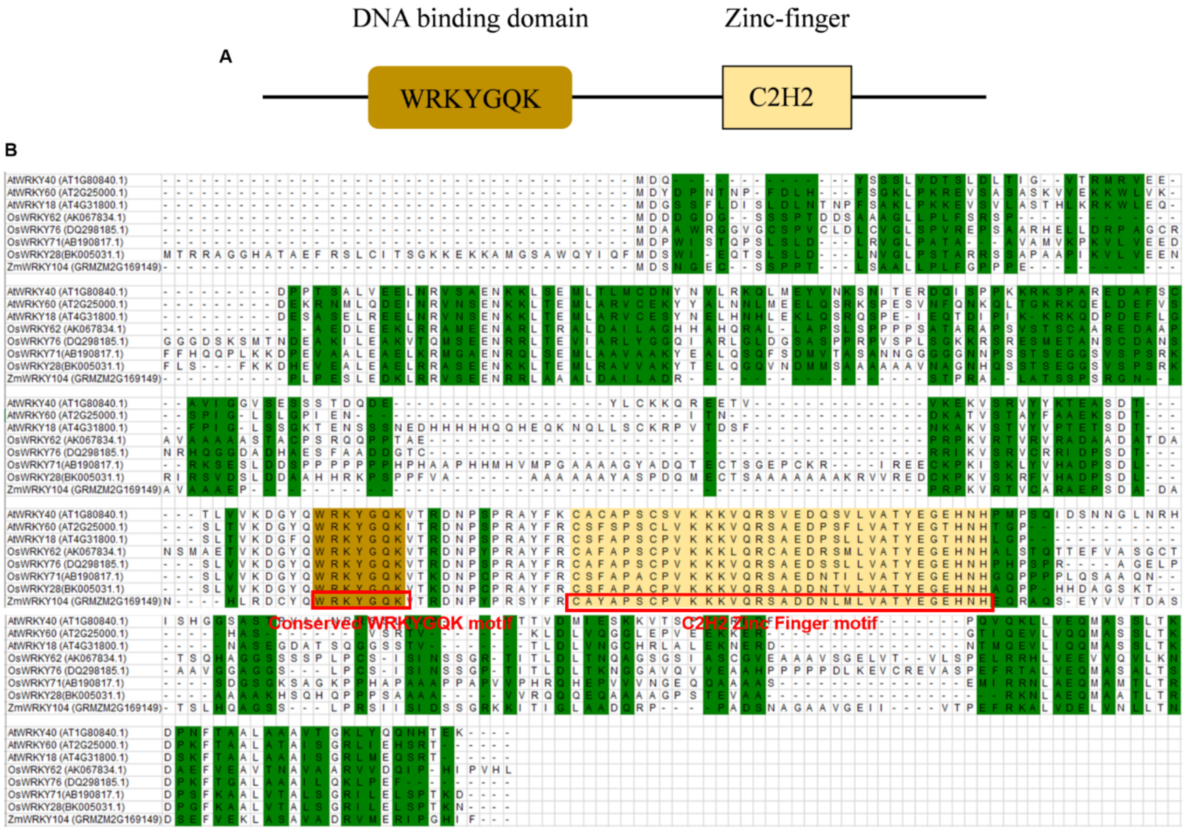

Figure S1. Multiple alignment of ZmWRKY104 with other orthologs in rice, *Arabidopsis*. (A) Conserved domains of ZmWRKY104. (B) Multiple alignment of ZmWRKY104 with other orthologs in rice, *Arabidopsis*. The first red box indicates the WRKYGQK motif, and the second indicates the conserved C2H2 zinc-finger motif. The accession numbers: AtWRKY40 (AT1G80840.1), AtWRKY60 (AT2G25000.1), AtWRKY18 (AT4G31800.1), OsWRKY62 (AK067834.1), OsWRKY76 (DQ298185.1), OsWRKY71(AB190817.1), OsWRKY28(BK005031.1), ZmWRKY104 (GRMZM2G169149). At, *Arabidopsis thaliana*; *Zm*, *Zea mays*; *Os*, *Oryza sativa*.

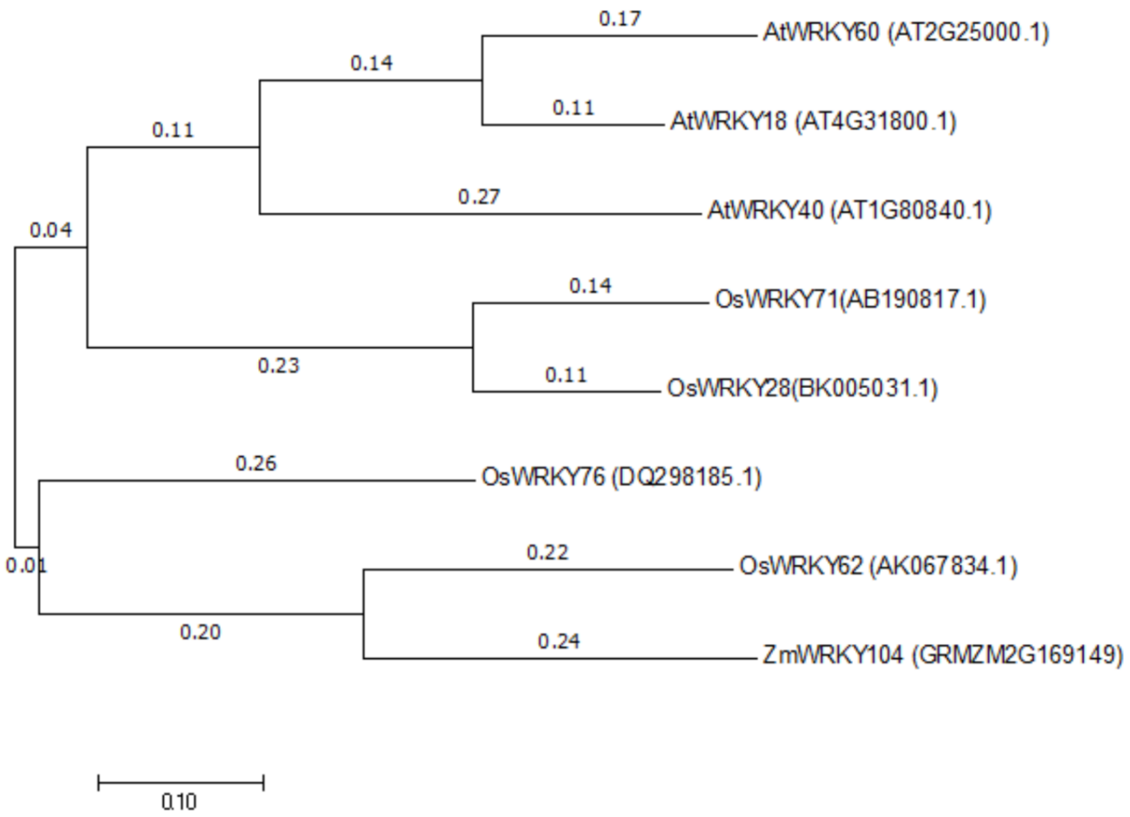

Figure S2. Phylogenetic relationships of ZmWRKY104 with other orthologs in rice, *Arabidopsis*. The amino acid sequences of WRKYs alignment were completed by ClustalW, and MEGA 7 was used to construct a phylogenetic tree using the neighbor-joining method.

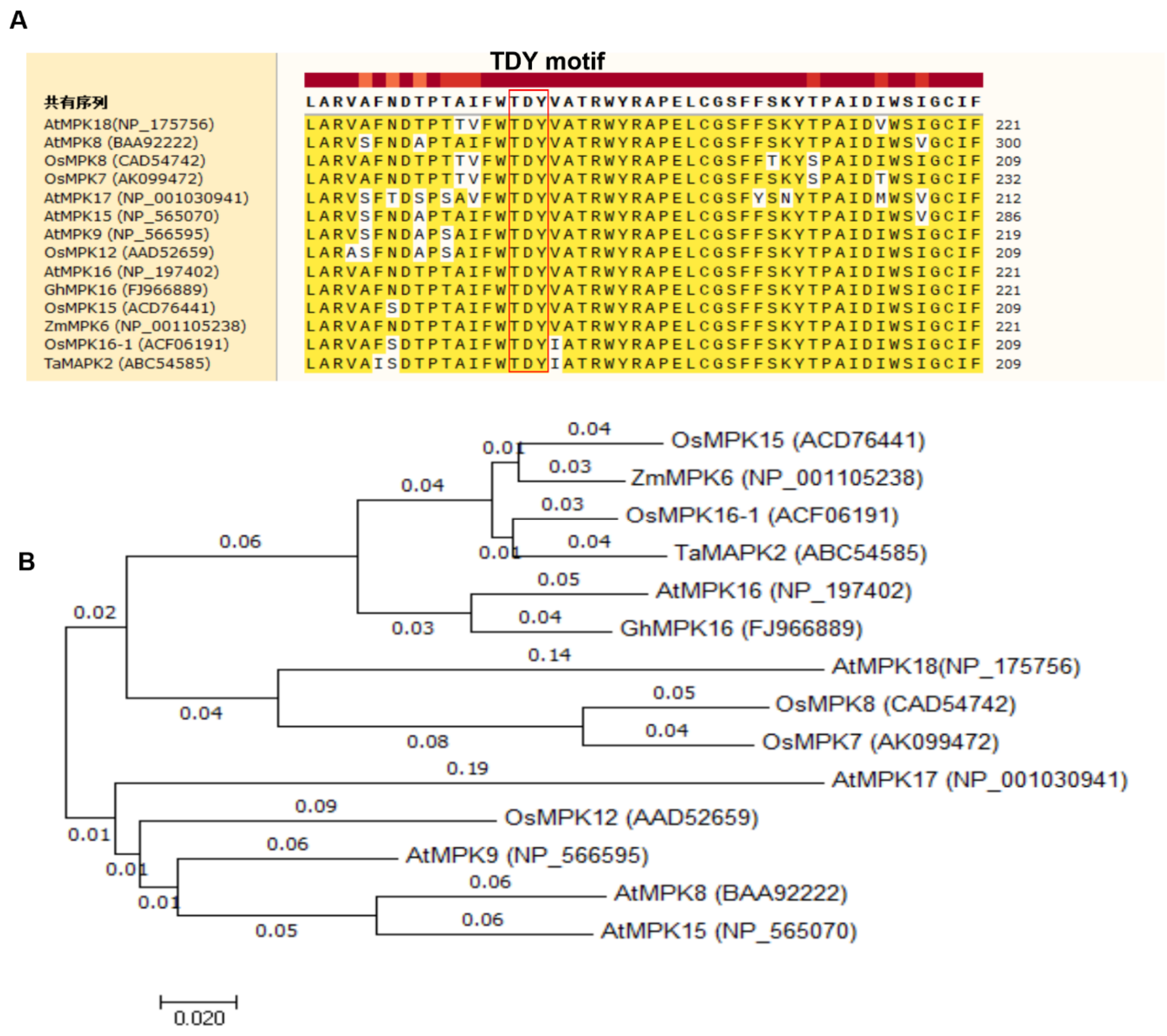

Figure S3. Multiple alignment and phylogenetic relationships of ZmMPK6 with other orthologs in rice, *Arabidopsis*, wheat, cotton. (A) Multiple alignment of ZmMPK6 with other orthologs in rice, *Arabidopsis*, wheat, cotton. Similar residues were shaded in yellow, respectively. The TDY activation motif was boxed in red. (B) Phylogenetic relationships of ZmMPK6 with other orthologs in different species. The amino acid sequences of MAPKs alignment were completed by ClustalW, and MEGA 7 was used to construct a phylogenetic tree using the neighbor-joining method. The accession numbers: AtMPK18 (NP_175756), AtMPK8 (BAA92222), OsMPK8 (CAD54742), OsMPK7 (AK099472), AtMPK17 (NP_001030941), AtMPK15 (NP_565070), AtMPK9 (NP_566595), OsMPK12 (AAD52659), AtMPK16 (NP_197402), GhMPK16 (FJ966889), OsMPK15 (ACD76441), ZmMPK6 (NP_001105238), OsMPK16-1 (ACF06191), TaMAPK2 (ABC54585). At, *Arabidopsis thaliana*; *Zm*, *Zea mays*; *Os*, *Oryza sativa*; *Ta*, *Triticum aestivum*; *Gossypium hirsutum*.

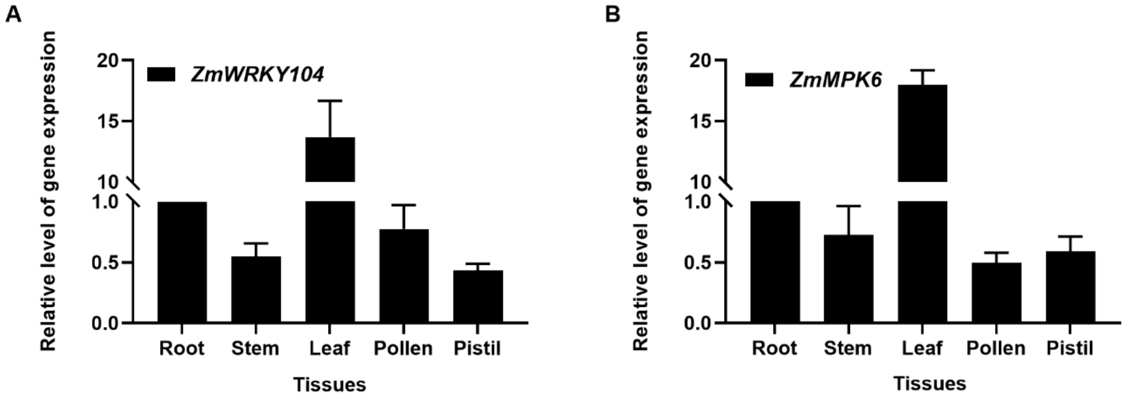

Figure S4. RT-qPCR analysis of *ZmWRKY104* and *ZmMPK6* expression in different tissues of maize. Total RNA was isolated from roots, stems, leaves, pollen, and pistil, the expression of (A) *ZmWRKY104* and (B) *ZmMPK6* relative to *ZmActin* was determined by RT-qPCR analysis. Data are the means ± SE (n = 3).

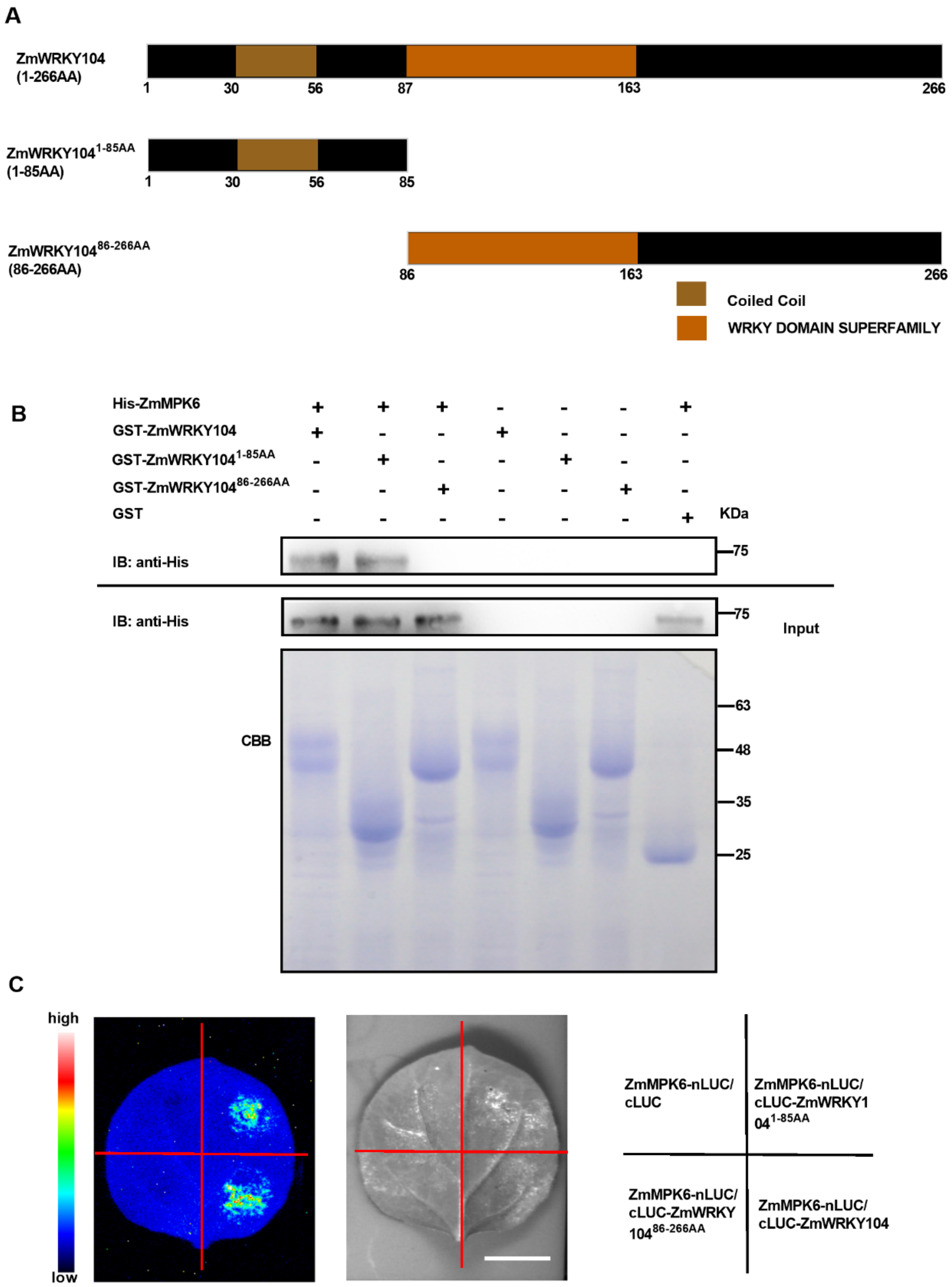

Figure S5. ZmMPK6 interacts with the truncations of ZmWRKY104. (A) The truncations of ZmWRKY104. ZmWRKY104$^{1\text{-}85AA}$ consists of 1-85 amino acids of the N-terminal of ZmWRKY104 protein, ZmWRKY104$^{86\text{-}266AA}$ consists of 86-266 amino acids of the C-terminal of ZmWRKY104 protein. (B) GST pull-down. GST-ZmWRKY104 fusion protein and its truncations (ZmWRKY104$^{1\text{-}}$

^{85AA}, ZmWRKY104$^{86\text{-}266AA}$) or GST alone was incubated with His-ZmMPK6 protein in GST beads. His-ZmMPK6 was detected by western blot using Anti-His antibody. GST-ZmWRKY104 fusion protein and its truncations or GST were then detected by SDS-PAGE. Molecular mass markers in kilodaltons are shown on the right. (C) LCI. *ZmMPK6* was fused to the nLUC, the full-length ZmWRKY104 protein and its truncations were fused to the cLUC， and then co-expressed in *Nicotiana benthamiana* leaves. ZmMPK6-nLUC/cLUC-ZmWRKY104 was used as a positive control, ZmMPK6-nLUC/cLUC was used as a negative control. Luciferase signals were captured using the Tanon-5200 image system. Scale bar, 1 cm. All the experiments were repeated at least three times with similar results.

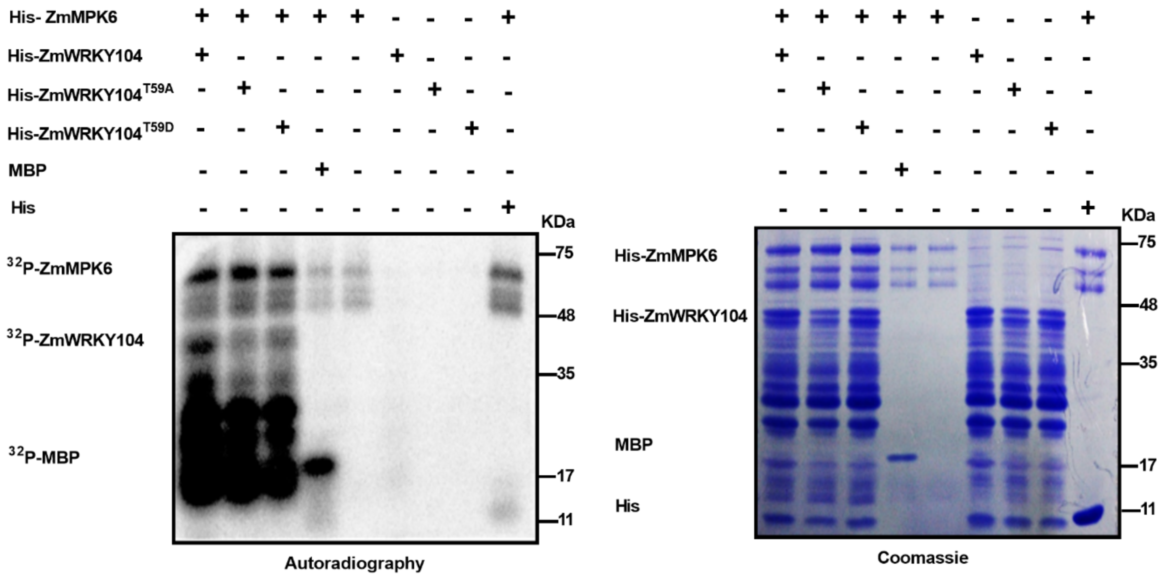

Figure S6. Phosphorylation of ZmWRKY104^{T59A} and ZmWRKY104^{T59D} by ZmMPK6 *in vitro*. His-ZmMPK6, His-ZmWRKY104, His-ZmWRKY104^{T59A}, His-ZmWRKY104^{T59D} and His protein are expressed in *E. coli*. *In vitro* kinase assays are performed using the purified proteins. Recombinant His-ZmWRKY104, His-ZmWRKY104^{T59A}, His-ZmWRKY104^{T59D} or maltose binding protein (MBP) are used as substrate and subjected to in-gel kinase assay. Images show autoradiography (left panel), the corresponding coomassie staining (right panel). Molecular mass markers in kilodaltons are shown on the right. All the experiments were repeated at least three times with similar results.

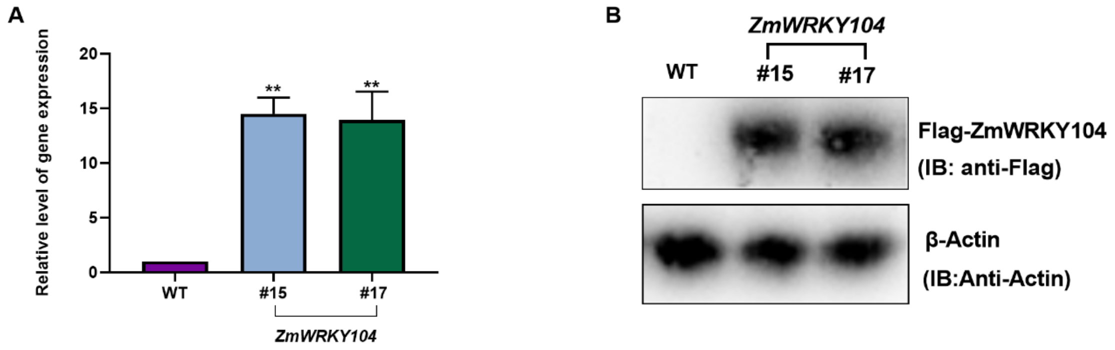

Figure S7. Analysis the gene expression and protein level of the *ZmWRKY104* transgenic lines. (A) The expression level of *ZmWRKY104* (#15, #17) in transgenic maize was analyzed by RT-qPCR. Values are the means ± SE of three different experiments. P values were calculated by the Student's *t* tests (**P <0.01). (B) Western blotting (WB) analysis of ZmWRKY104 (#15, #17) protein, ZmWRKY104 was analyzed by immunoblotting (IB) using an Anti-Flag antibody; β-actin was used as the total protein loading control.

Table S1. PCR Primers Used

Primer name	Vector	Primer sequence (5’–3’)
**Primers for gene clone**		
ZmWRKY104-F		ATGGACAGTAACGGCGAGTGC
ZmWRKY104-R		CTAGAATATGTGCCCTGGTATTCTC
**Primers for Y2H**		
AD-ZmWRKY104-F	pGADT7	GAATTCATGGACAGTAACGGCGAGTGC
AD-ZmWRKY104-R	pGADT7	GGATCCCTAGAATATGTGCCCTGGTATTCT
BD-ZmMPK6-F	pGBKT7	GGATCCATGCAGCACGACCAGAAGAAGAAGGCACC
BD-ZmMPK6-R	pGBKT7	CTGCAGCTACCAGTGACCACCACGACTATCAACGGT
**Primers for GST-pull down**		
GST-ZmWRKY104-F	pGEX-4T-1	GGATCCATGGACAGTAACGGCGAGTG
GST-ZmWRKY104-R	pGEX-4T-1	GAATTCGAATATGTGCCCTGGTATTCTC
GST-ZmWRKY104$^{1\text{-}85AA}$*-F*	pGEX-4T-1	GGATCCATGGACAGTAACGGCGAGTG
GST-ZmWRKY104$^{1\text{-}85AA}$*-R*	pGEX-4T-1	GAATTCCCGGACCTTGGGCCG
GST-ZmWRKY104$^{86\text{-}266AA}$*-F*	pGEX-4T-1	GGATCCATGACGGTGTGCGCGC
GST-ZmWRKY104$^{86\text{-}266AA}$*-R*	pGEX-4T-1	GAATTCGAATATGTGCCCTGGTATTCTC
His-ZmWRKY104-F	pET-30a	GGATCCATGGACAGTAACGGCGAGTG
His-ZmWRKY104-R	pET-30a	GAATTCGAATATGTGCCCTGGTATTCTC
His-ZmMPK6-F	pET-30a	GGATCCATGCAGCACGACCAGAAGAAGAAGGC
His-ZmMPK6-R	pET-30a	GAGCTCCCAGTGACCACCACGACTATCAACGGT
**Primers for LCI**		
ZmMPK6-nLUC-F	pCAMBIA1300	GAGCTCATGCAGCACGACCAGAAGAAG

ZmMPK6-nLUC-R	pCAMBIA1300	GGATCCCCCCAGTGACCACCACGACTATC
ZmWRKY104-cLUC-F	pCAMBIA1300	GGTACCATGGACAGTAACGGCGAGT
ZmWRKY104-cLUC-R	pCAMBIA1300	GGATCCGAATATGTGCCCTGGT
ZmWRKY104^{1-85AA}*-cLUC-F*	pCAMBIA1300	GGTACCATGGACAGTAACGGCGAG
ZmWRKY104^{1-85AA}*-cLUC-R*	pCAMBIA1300	GGATCCCCGGACCTTGGGCCGCGG
ZmWRKY104$^{86-266AA}$*-cLUC-F*	pCAMBIA1300	GGTACCATGACGGTGTGCGCGCGC
ZmWRKY104$^{86-266AA}$*-cLUC-R*	pCAMBIA1300	GGATCCGAATATGTGCCCTGGTAT

Primers for Co-IP

*ZmWRKY104-3*flag-F*	1300-221-3*flag	GGTACCATGGACAGTAACGGCGAGTG
*ZmWRKY104-3*flag-R*	1300-221-3*flag	GGATCCTCAGAATATGTGCCCTGGTATTCTC
*ZmMPK6-6*myc-F*	1300-221-6*myc	TTCGAAATGCAGCACGACCAGAAGA
*ZmMPK6-6*myc-R*	1300-221-6*myc	GGATCCCTACCAGTGACCACCACGA

Primers for qRT

ZmMPK6-qRT-F		CAGAAGAAGCCAGACCTGCA
ZmMPK6-qRT-R		CCACCACGACTATCAACGGT
ZmWRKY104-qRT-F		TCCGAGATCCTACTTCCGCT
ZmWRKY104-qRT-R		ATATTCGCTCTGGGCACGTT
ZmActin2-qRT-F		GCCATCCATGATCGGTATGG
ZmActin2-qRT-R		GTCGCACTTCATGATGGAGTTG

Primers used in subcellular localization

ZmWRKY104-YFP-F	1305-d35SYFP	GGTACCATGGACAGTAACGGCGAGTG
ZmWRKY104-YFP-R	1305-d35SYFP	GGATCCTCAGAATATGTGCCCTGGTATTCTC
ZmMPK6-YFP-F	1305-d35SYFP	TCTAGAATGCAGCACGACCAGAAGAAGAAGGCA

ZmMPK6-YFP-R	1305-d35SYFP	GGATCCCCAGTGACCACCACGACTATCAACGGTAG

Primers for dsRNA

ds-ZmWRKY104-F		TAATACGACTCACTATAGGGGCCATACTCGCCGACCGCT
ds-ZmWRKY104-R		TAATACGACTCACTATAGGGCTGGCCGTACTTGCGCCAC

Primers used in maize protoplasts

Ubi: ZmWRKY104-mcherry-F	pXZP008	GGATCCATGGACAGTAACGGCGAGTG
Ubi: ZmWRKY104-mcherry-R	pXZP008	GGTACCACGAATATGTGCCCTGGTATTCTC
Ubi: ZmWRKY104-SRDX-mcherry-F	pXZP008	GGATCCATGGACAGTAACGGCGAGTG
Ubi: ZmWRKY104-SRDX-mcherry-R	pXZP008	GGTACCGTCAAACGGAGTTCTAGATCCAGGAATATGTGCCCTGGTATTCTC
Ubi: ZmMPK6-mcherry-F	pXZP008	TCTAGAATGCAGCACGACCAGAAGAAGAAGGCAC
Ubi: ZmMPK6-mcherry-R	pXZP008	GGATCCCCAGTGACCACCACGACTATCAACGGT

Primers used in transgenic maize

pCUN - ZmWRKY104-F	pCUN-N-HF	AAGCTTAATGGACAGTAACGGCGAG
pCUN - ZmWRKY104-R	pCUN-N-HF	GGTACCCTAGAATATGTGCCCTGGTATTCT
Primer 1-F		ATCGAGACAAGCACGGTCAA
Primer 1-R		AAACCCACGTCATGCCAGTT
Primer 2-F		GGACGATGACGATAAGCTGGAAGT
Primer 2-R		CATCAGATTGTCGTCTGCGCTT

F, forward primer; R, reverse primer.

DNA oligos used for site-directed mutangenesis

**Oligo Name (Gene-mutation)**	**Oligo for mutagenesis**
ZmWRKY104-T59A	TCGCCGACCGCTCCGCCCCGCGAGCGCTCG
ZmWRKY104-T59D	TCGCCGACCGCTCCGACCCGCGAGCGCTCG
